# A Feasibility Study on a Portable Vision Device for Patients with Stroke and Brain Tumours

**DOI:** 10.1080/01658107.2023.2273471

**Published:** 2024-02-12

**Authors:** Arina Nisanova, Laurel Barrios, Tanvi Chokshi, Mark Mannis, Orin Bloch, Yin Allison Liu

**Affiliations:** a School of Medicine, University of California, Davis, California, USA; b College of Medicine, California Northstate University, Elk Grove, California, USA; c Department of Ophthalmology & Vision Science, University of California, Davis, California, USA; d Department of Neurological Surgery, University of California, Davis, California, USA; e Department of Neurology, University of California, Davis, California, USA

**Keywords:** Low vision, portable vision device, vision-related quality of life, stroke, brain tumour, visual field defect

## Abstract

This prospective, single-centre cohort study aimed to evaluate the impact of a portable vision reading device, OrCam Read, on vision-related quality-of-life and independent functional status in patients with low vision due to stroke or brain tumours. Six patients with poor visual acuity or visual field defects due to a stroke or a brain tumour were enrolled at a U.S. Ophthalmology Department. Participants were trained to use OrCam Read and given a loaner device for the 1 month duration of the study. Various assessments, including daily function tests, the National Eye Institute Visual Function Questionnaire-25, and the 10-item neuro-ophthalmic supplement, were administered at the first and last visits. Patients’ experience with the device was evaluated with weekly telephone and end-of-study satisfaction surveys. The main outcome measures were the patient satisfaction with OrCam and the mean assessment scores between enrolment and final visits. The intervention with OrCam significantly improved patients’ ability to complete daily tasks and participants reported good satisfaction with the device. The results also show non-significant improvement with distant activities, dependency, and role difficulties. Our findings demonstrate the feasibility of studying vision-related quality-of-life using a portable vision device in this patient population and pave the way for a larger study to validate the results of this study.

## Introduction

Low vision is defined as permanent visual impairment with best corrected visual acuity (BCVA) worse than 20/70 and better than 20/400, substantial visual field loss, or substantial loss of contrast sensitivity in the better-seeing eye, and that the condition cannot be corrected by refraction, medical treatment or surgery.^1,2^ According to the projections based on the U.S. census data, more than 8 million Americans are expected to suffer from visual impairment or blindness.^3^ Low vision is associated with an increased risk of depression^4,5^ and injuries^6,7^ and is the third-ranked condition that requires assistance with daily activities in people older than 70-years-old.^1^

Stroke and brain tumours frequently result in visual impairments^8–10^ and have a profound impact on quality-of-life (QoL).^8,11^ The latter has become an increasingly relevant area of clinical neuro-oncology research as survivorship increases.^12^ Neuro-oncology patients frequently experience visual symptoms associated with the tumours and their treatment.^13–16^ Patients with low vision face varying degrees of limitations in their daily functions and must rely on the help of others or vision aids to read, perform routine activities, and orient themselves.^1,11,17,18^ In the absence of curative treatment, low vision rehabilitation, optical devices, and vision aids serve as useful tools to help patients read and perform daily activities. There is no gold standard approach in treating low vision and the evidence regarding vision aid efficacy is conflicting. Some reports showed that they improved independent functional status,^11,19^ increased reading speed,^20,21^ and positively affected well-being,^21^ while others failed to find any added value.^22^

OrCam Read is a handheld device powered by artificial intelligence and a smart camera that captures printed or digital text and plays it out loud. Previous reports demonstrated that the use of the other version of the device, OrCam MyEye, improved vision-related QoL (VRQoL) and the ability to perform daily tasks in patients with glaucoma-related^23^ and other causes of low vision.^24^ The impact of vision devices on QoL in patients with stroke and brain tumour-related low vision has not been evaluated before. In the present feasibility study, we report the impact of using OrCam Read on the QoL in patients with low vision as a result of a stroke or a primary brain tumour. Investigation among this population may shed light on the effect of this device on the patients’ self-reported QoL and daily functioning.

## Materials and methods

This was a prospective feasibility study that included patients with poor visual acuity or severe visual field defects affecting their ability to read. The study was conducted in accordance with the Declaration of Helsinki. The Institutional Review Board (IRB #1704635–2 and #1704618–2) approved the study protocol at the University of California, Davis. All participants signed an informed consent document before enrolment.

### Study population

Between March and September 2021, we screened 10 consecutive patients with low vision, either due to haemorrhagic/ischaemic stroke or a primary brain tumour, who received neuro-ophthalmic care at the University of California, Davis. Six patients were eligible and consented for the study. The enrolment criteria included: age older than 21 years with BCVA between hand motion and 20/400 in the better-seeing eye and/or visual field defects (VFDs) in one or both eyes, resulting in difficulty reading. All participants were fluent English speakers, capable of understanding text-to-speech auditory readings and using a computerised system. Exclusion criteria included patients with: 1) low vision unrelated to stroke or brain tumour; 2) ophthalmic conditions before stroke or tumour diagnosis such as glaucoma, macular degeneration, diabetic retinopathy, or abnormal visual fields determined by perimetry; 3) inability to operate the device due to cognitive impairment, hand disability, deafness or severe hearing impairment; and 4) poor text-to-speech comprehension.

### The OrCam Read device

**Figure 1.**
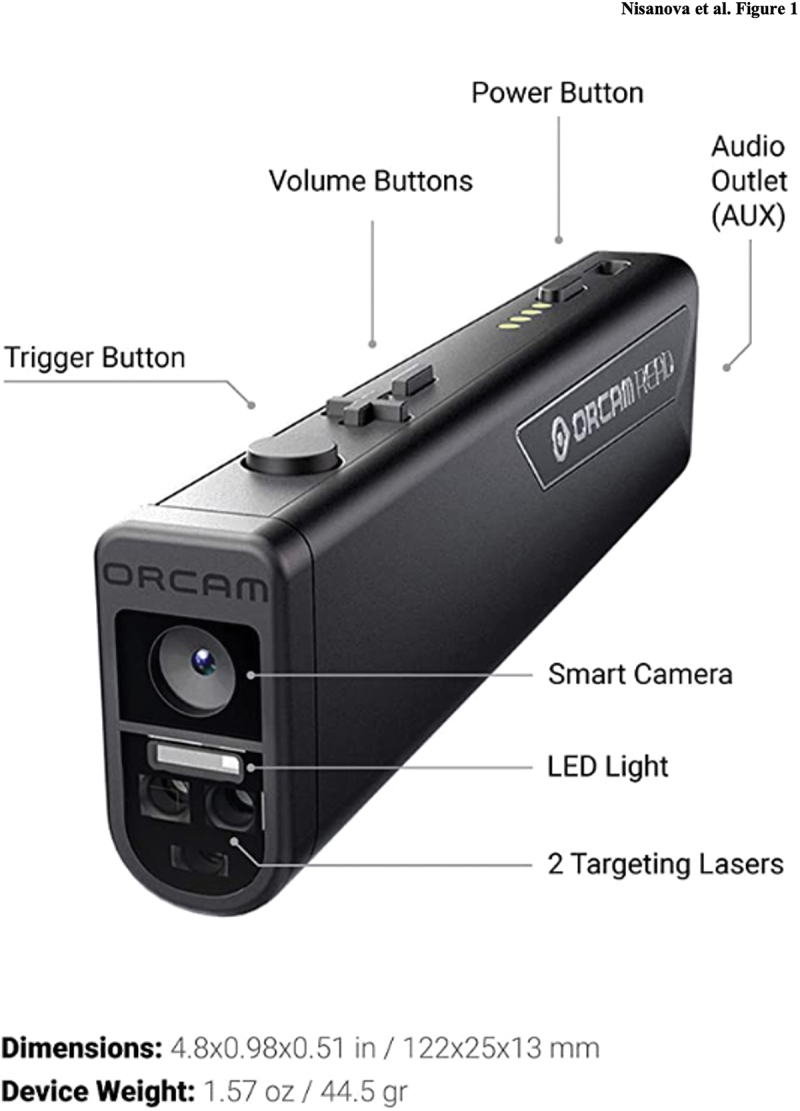
OrCam read. OrCam read is a handheld, Bluetooth-connected device powered by artificial intelligence equipped with a smart camera capable of capturing printed or digital text and playing it out loud.

This OrCam Read unit includes a small, handheld device equipped with a visual sensor powered by artificial intelligence and computer vision that can capture text from any surface, including digital and printed (Figure 1). It has a light emitting diode light for poorly lit environments and two laser modes for capturing the text. The device is both button and voice-controlled, allowing the user to specify the amount of text desired to be read. The user can activate the device by saying ‘Hey OrCam’ and control the reading, text navigation, and settings. OrCam Read is advertised as an aid for people with low vision, reading and learning disabilities, or anyone who consumes large amounts of text.

AUX = auxiliary; LED = light emitting diode.

### Study design

This 1 month prospective cohort feasibility study consisted of two in-person visits (at enrolment and at conclusion of the study) and three weekly telephone visits. The study timeline is shown in Table 1. At the screening/initial visit, we recorded patients’ BCVA using the Snellen chart. The participants were provided with the OrCam Read device and a 90-to-120-minute training session on the use of the device by an experienced clinical trial coordinator to perform daily tasks in the daily function test (DFT) (Table 2). At the enrolment visit, these tasks were performed with the participants’ best assisted and corrected vision. All participants also completed the 25-item National Eye Institute Visual Function Questionnaire (NEI-VFQ-25) with a 10-item neuro-ophthalmic supplement (10-NOS) at the enrolment visit. The participants were encouraged to use the OrCam Read device in their daily living as much as possible.

**Table 1.** Study timeline.

Study Procedure	Consent	Device training	Daily function test	NEI-VFQ-25	10-item NOS	Weekly 4-item telephone questionnaire	Satisfaction survey
Time Estimate	30 minutes	90 to 120 minutes	30–45 minutes	30 minutes	20 minutes	20 minutes	15 minutes
Screening visit	x	x					
Study visit #1			x	x	x		
Day after tech support/virtual meeting (week 1)						x	
Day after tech support/virtual meeting (week 2)						x	
Day after tech support/virtual meeting (week 3)						x	
Study visit #2			x	x	x		x

10-item NOS = 10-item Neuro-Ophthalmic Supplement NEI-VQF-25 = National Eye Institute Visual Function Questionnaire-25.

**Table 2.** Daily function tasks descriptions, instructions, and mean scores at baseline and after a 1-month intervention with OrCam read.

	Baseline	Follow-up
Task & Instructions	Number (%) who completed the task	
1. Read a printed letter. *Patients are given an envelope with a single-page letter enclosed and asked to read it aloud.*	2 (40%)	5 (100%)
2. Read a menu. *Patients are given a local restaurant menu and asked to read it aloud and make a selection.*	3 (60%)	4 (80%)
3. Read a newspaper article. *Patients are given a piece of newspaper and asked to read aloud a specific article.*	2 (40%)	5 (100%)
4. Read an email message on an electronic device. *Patients are handed a device and asked to read aloud the content of the email.*	2 (40%)	5 (100%)
5. Read a text message on the mobile phone. *Patients are handed a smartphone and asked to read aloud a text message displayed on it.*	1 (20%)	4 (80%)
	Mean Score (standard deviation)	
**Total Daily Function Test Score**	2.5 (1.7)	4.6 (0.9)
	**p = .0495**	

The subjects then attended three weekly virtual group meetings between the baseline and the final visits. The meetings were hosted by the OrCam team and were designed to provide technical support and collect feedback. We conducted three weekly telephone surveys 1 day after the group meeting. The surveys consisted of a three-item questionnaire including: ‘1) How easy was it to use OrCam? 2) Is the technical support helpful? 3) How much time they were using the OrCam device daily?’ This was to monitor the participants’ progress and evaluate the ease and usefulness of the device.

At the final visit, all participants were asked to perform the tasks in the daily function test with the assistance of the OrCam Read Device in addition to the patients’ routine visual assistance methods if participants elected to use them. NEI-VFQ-25 and 10-NOS were repeated, and participants also completed a satisfaction survey to assess their experience and provide feedback about the device.

### Study questionnaires

The DFT assessed the subjects’ performance on daily tasks and gave them credit if they performed five tasks before and after the study period with OrCam: read a printed letter; a menu; a newspaper article; an email; and a text message (Table 2). In a previous study,^24^ similar tasks were used to assess the improvement of difficulties in daily reading needs for patients with low vision using a previous version of the OrCam device. The participants’ performance was recorded by a clinical research coordinator, and for each task, a score of 1 was given if the participant completed the task and 0 if not, yielding a total score of 0 to 5 for each test.

The NEI-VFQ-25 is a validated and reliable tool widely used to assess self-reported vision-related QoL.^25^ The 10-NOS is a supplemental, validated series of questions that increases the capacity of the NEI-VFQ-25 to capture patient-reported visual dysfunction in patients with neuro-ophthalmologic disorders.^26^ The participants were encouraged to use the OrCam Read device in their regular settings. The satisfaction survey was developed based on other previous studies ^23,24^ to describe their experience with the device and provide feedback.

### Statistical analysis

We used GraphPad Prism 9 (San Diego, CA, version 9.5.0) and Microsoft Excel (Seattle, WA, version 16.68) to analyse the data and generate descriptive statistics. We used the unpaired t-test to evaluate the significance of the mean test scores with and without the OrCam Read device at different time points. The findings were considered significant if the *p*-value was less than .05.

## Results

Ten potential participants with low vision were screened prior to enrolment. One patient was excluded due to a pre-existing ophthalmic condition, two due to low vision unrelated to stroke of brain tumour, and one patient met the inclusion criteria but did not wish to participate. Six patients participated in this feasibility study. The patient characteristics are summarised in Table 3. Four (67%) participants were female and two were male (33%). The mean age at enrolment was 62.8 ± 17.8 years (range 40 to 83). The aetiology of low vision was stroke in three patients and a primary brain tumour in the other three patients. The locations of the brain injuries were right parieto-occipital, left temporal, or left occipital lobe in patients who had suffered a stroke. The other three patients had the following tumours: anaplastic astrocytoma located in the right temporal lobe; sellar/suprasellar/cavernous sinus meningioma; and the World Health Organization (WHO) grade 4 glioblastoma in the left occipito-temporal lobe. One patient had severe vision loss, and the other five were enrolled based on severe visual function defects causing difficulty reading. All participants completed all study tasks except for two patients who did not complete the final NEI-VQF-25 and another patient with glioblastoma who passed away shortly after enrolment and was unable to complete weekly check-ins and the final DFT, NEI-VQF-25, 10-item NOS, and satisfaction surveys.

**Table 3.** Participant demographic and clinical characteristics.

Mean age (years, standard deviation)		62.8 (17.83)
Sex	Female	4 (67%)
	Male	2 (33%)
Aetiology		
Brain tumour (*n* = 3), location	Anaplastic astrocytoma, right temporal lobe	1 (17%)
	Meningioma, sella/suprasellar/cavernous sinus	1 (17%)
	Glioblastoma, left occipito-temporal lobe	1 (17%)
Stroke (*n* = 3), location	Temporal lobe	1 (17%)
	Parietal lobe	1 (17%)
	Occipital lobe	1 (17%)
Visual acuity (median, range)	Better eye Snellen BCVA	20/30 (HM to 20/20)
	Worse eye Snellen BCVA	20/45 (NLP to 20/20)
Low vision impact	BCVA <20/400	1 (17%)
	Homonymous hemianopia	5 (83%)
Survival throughout the study	Alive	5 (83%)
	Deceased	1 (17%)

BCVA = best corrected visual acuity; HM = hand motion; NLP = no light perception.

### BCVA, NEI-VFQ-25, and 10-item NOS

The median (range) Snellen BCVA at baseline was 20/30 (range: hand motion [HM] to 20/20) in the better-seeing eye and 20/45 (range: no light perception [NLP] to 20/20) in the worse eye. The median Early Treatment of Diabetic Retinopathy Study score was 80.5 (range: 5 to 89) in the better eye and 79 (range: NLP to 89) in the worse eye. We calculated the NEI-VFQ-25 scores as instructed in the questionnaire’s manual. We converted the raw, Likert-like score into a number from 0 to 100, with 100 indicating fully comfortable with each test question or task. The scores per each of the 13 subscales were then averaged (Table 4). The subscale scores (excluding the General Health subscale) were further averaged to generate the composite score. The 10-NOS scores were calculated in a similar manner. The scores ranged from 0 to 100, and a higher score was indicative of a better QoL or VRQoL. The mean (standard deviation) composite NEI-VQF-25 score was 44.3 (11.8, *n* = 6) at baseline and 41.7 (SD 8.4, *n* = 3) at the final visit. The difference between the means was found to be not significant (*p* = .8). The mean composite 10-item NOS score was 41.7 (20.5, *n* = 6) at baseline and 47.1 (13.3, *n* = 5) at the final visit. The difference between the mean composite 10-item NOS scores was also found to be not significant (*p* = .63). The subscale NEI-VQF-25 and 10-item NOS scores are summarised in Table 4. None of the subscales showed significant changes on VRQoL. The greatest average score improvement on the NEI-VQF-25 was seen in the vision-specific dependency subscale from baseline (23.6) to follow-up (38.9, *p* = .22). The greatest average score improvement on the 10-item NOS was observed in using the computer from baseline (33.3) to follow-up (45.0, *p* = .55) and difficulty in bright sunlight (54.2 to 70.0, *p* = .34). The greatest average score decrease on the NEI-VQF-25 was seen in the general vision subscale from baseline (60.0) to follow-up (46.7, *p* = .42). The greatest average score decrease on the 10-item NOS was observed in Item 9, which included binocular double vision (58.3 to 35.0, *p* = .37).

**Table 4.** Distribution of national eye institute visual function questionnaire-25 score changes including the 10-item neuro-ophthalmic supplement by categories before and after the 1 month intervention with OrCam read.

	Baseline mean (SD), median score	Follow-up mean (SD), median score	Mean score difference	p value
**NEI-VFQ-25 Subscales**				
General health	50 (22.4), 50	66.7 (28.9), 50	16.7	.37
General vision	60 (21.9), 60	46.7 (23.1), 60	−13.3	.42
Ocular pain	81.3 (19), 81.3	75 (12.5), 75	−6.3	.63
Near activities	36.1 (11.4), 33.3	30.6 (17.3), 25	−5.6	.58
Distance activities	50.7 (23.9), 58.3	63.9 (17.3), 58.3	13.2	.43
Vision specific: Dependency	23.6 (15.3), 20.8	38.9 (17.3), 33.3	15.3	.22
Vision specific: Mental health	24 (21.1), 28.1	25 (28.6), 18.8	1.0	.95
Vision specific: Role difficulties	18.8 (22), 12.5	33.3 (19.1), 37.5	14.6	.36
Vision specific: Social functioning	66.7 (31.3), 68.8	62.5 (33.1), 50	−4.2	.86
Driving	0 (0), 0	0 (0), 0	0.0	
Colour vision	70.8 (36.8), 75	58.3 (28.9), 75	−12.5	.63
Peripheral vision	37.5 (30.6), 25	25 (25), 25	−12.5	.56
Composite NEI-VQF-25 score	44.3 (11.8), 40.3	41.7 (8.4), 43.1	−2.5	.75
**10-item NOS**				
Item 1: Difficulty when eyes tired	45.8 (24.6), 50	50 (30.6), 50	4.2	.81
Item 2: Difficulty in bright sunlight	54.2 (29.2), 50	70 (20.9), 75	15.8	.34
Item 3: Difficulty parking car	0 (0), 0	0 (0), 0	0.0	
Item 4: Difficulty using computer	33.3 (30.3), 37.5	45 (32.6), 50	11.7	.55
Item 5: Two eyes see differently	20.8 (40.1), 0	30 (41.1), 25	9.2	.72
Item 6: Eye/lid appearance unusual	70.8 (45.9), 100	80 (32.6), 100	9.2	.72
Item 7: Vision blurry, not clear, ‘fuzzy’	20.8 (33.2), 0	15 (22.4), 0	−5.8	.75
Item 8: Trouble focusing on moving objects	41.7 (40.8), 37.5	50 (39.5), 50	8.3	.74
Item 9: Binocular double vision	58.3 (40.8), 62.5	35 (41.8), 25	−23.3	.37
Item 10: Ptosis	70.8 (45.9), 100	75 (35.4), 100	4.2	.87
Composite 10-item NOS score	41.7 (20.5), 43.8	47.1 (13.3), 47.5	5.4	.63

10-item NOS = 10-item neuro-ophthalmic supplement; NEI-VQF-25 = National Eye Institute Visual Function Questionnaire-25; SD = standard deviation.

### Weekly check-ins

**Figure 2.**
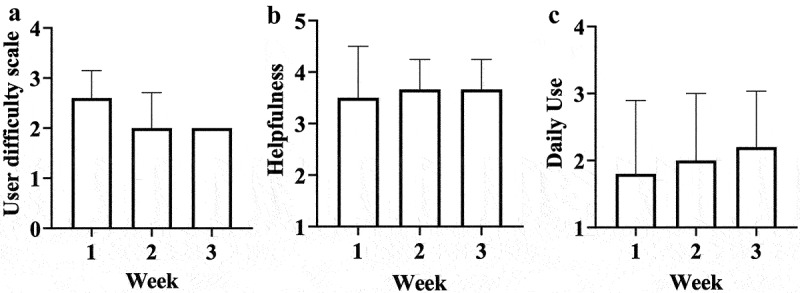
Weekly telephone check-in data over a 3-week period. (a) Participants were asked how easy OrCam was to use: 1 = very easy; 2 = easy; 3 = difficult; 4 = very difficult. b) Participants were asked to rate how helpful they found the virtual support meetings/groups to be: 1 = not at all; 2 = somewhat helpful; 3 = helpful; 4 = extremely helpful. (c) Participants were asked how long they spent using OrCam daily: 1 = less than 30 minutes; 2 = 30 to 60 minutes; 3 = 1 to 2 hours; 4 = more than 2 hours.

The check-in results are shown in Figure 2. Five patients completed the weekly check-ins (83%); one patient (17%) did not complete the phone visits. Out of the five participants, four (80%) reported using OrCam from 30 minutes to 2 hours on average per day. Patients reported the device to be easy (40%) or difficult (60%) to use at the first check-in, but everyone found it easy to use by the third weekly check-in. Four patients (67%) found the virtual support meetings helpful; one patient did not attend them. One participant remarked that ‘OrCam was so good, it made me cry’.

### Daily function tests

The DFT prompts and results are summarised in Table 2. At baseline, five (83%) patients had low vision defined by homonymous hemianopia and reported difficulty reading due to this impairment. The participants could complete an average (SD) of 2.5 (1.7) out of 5 tasks assessed on the daily function test with their best-corrected vision. At the follow-up visit, participants used the OrCam device in addition to a preferred method of visual assistance, and the mean number of tasks performed was 4.6 (0.9). The mean difference in DFT scores was 2.1 (*p* = .0495). The most challenging task was reading a text message on a smartphone. Only one (20%) patient was able to read a smartphone text at baseline versus four (80%) at follow-up out of five patients that completed DFTs.

### Satisfaction survey

Five patients completed the satisfaction survey (83%) (Table 5). Of the five participants, 80% found the OrCam Read device easy to use and to have improved their VRQoL. On average, it took participants one week to become comfortable with the device, and everyone found the virtual support meetings helpful. All patients reported using the device for reading printed materials, and three also used it for digital texts. On average, patients were likely to consider using OrCam in their daily lives, and four (80%) were ‘likely’ or ‘very likely’ to recommend the device to other visually impaired people.

**Table 5.** Satisfaction survey results after a 1 month intervention with OrCam read.

Question	Number (%)
1. How long did it take you to become comfortable with the device?	
1–3 days 1 week 2 weeks 3 weeks	2 (40%) 1 (20%) 1 (20%) 1 (20%)
2. How easy was OrCam to use?	
Very easy Easy Difficult Very difficult	0 (0%) 4 (80%) 1 (20%) 0 (0%)
3. Were the technical support/virtual group meetings helpful?	
Not at all Somewhat Yes Yes, extremely	0 (0%) 1 (20%) 2 (40%) 1 (20%)
4. On average, how much did you spend using our OrCam per day?	
Less than 30 minutes 30 minutes to 1 hour 1 to 2 hours More than 2 hours	2 (40%) 2 (40%) 1 (20%) 0 (0%)
5. What did you find helpful using the OrCam device?	
Reading text from printed materials Reading digital texts Product recognition Face recognition Color recognition Reading signs Money recognition	5 (100%) 3 (60%) 0 (0%) 1 (20%) 0 (0%) 0 (0%) 0 (0%)
6. Overall, would you say that OrCam improved your vision-related quality-of-life?	
Made life difficult No Somewhat Yes	0 1 (20%) 2 (40%) 2 (40%)
7. How likely would you consider using OrCam in your daily life?	
Absolutely not Not likely Likely Very likely	0 2 (40%) 2 (40%) 1 (20%)
8. How likely would you recommend OrCam to another visually impaired person?	
Very unlikely Neutral Likely Very likely	0 1 (20%) 2 (40%) 2 (40%)

## Discussion

Our study demonstrated the feasibility of investigating the impact of a portable vision device on vision-related QoL in patients with visual impairment due to stroke or brain tumours, especially when they had difficulty reading due to VFDs. Most participants used the OrCam Read device from 30 min to 2 hours per day and felt comfortable using it within the first week of training. The NEI-VFQ and 10-NOS demonstrated that the OrCam Read device was helpful for computer use, decreasing role limitation in daily living due to low vision and improving independence in our participants. Easily accessible technical support was preferred by the participants, and most of them would recommend this device to visually impaired individuals.

Low vision contributes to impairments in QoL and affects patients’ daily activities, independence, and socio-emotional well-being.^27,28^ VFDs also affect reading performance by reducing reading speed and increasing reading time. Many existing aids, such as magnifiers or telescopes, assist patients by potentiating their remaining vision to help patients read or recognise objects,^29^ but they may not always offer additional benefits to patients with VFDs. Many visual rehabilitation methods have been studied to improve reading ability for patients with VFDs, especially hemianopia, such as changing the reading direction from horizontal to vertical^30^ and visual stimulation training.^31^ As such, differences in reading ability or performing daily activities following the incorporation of visual aids can be evident over a short period of time. Overall, after training and 1 month of use, patients were able to complete an average of four to five out of five tasks on the DFT using OrCam.

Although our study did not show statistically significant changes in the QoL on the NEI-VQF-25 and 10-item NOS, patients’ average scores improved in several domains. OrCam appeared to have a non-significant improvement with distant activities, dependency, and role difficulties. Mean scores indicated better self-reported general health, less need to rely on help from other people, and being less limited by vision when performing work or other activities. Patients also appeared to have less difficulty using computers. However, the mean scores decreased in several domains, including general, colour, peripheral, blurry, and double vision, as well as ocular pain and near activities. These findings are likely secondary to the progressive vision loss not alleviated by the use of the device, as well as limited follow-up data availability due to patients’ overall functional decline. The results of the satisfaction survey showed that OrCam had a positive impact on the participants’ VRQoL, although it should be noted that attending the weekly meetings might have also enhanced satisfaction scores. Overall, our results indicate that patients with low vision, regardless of the aetiology of visual impairment, may benefit from the device in completing daily activities independently, reading, or using personal technological devices.

Most participants found it easy to use, became comfortable with the device within one week, and were likely to consider using the device in their daily lives or recommending it to another visually impaired person. The device is handheld and can easily be manoeuvred by the patient. It can play the text through a built-in speaker, hard-wired headphones, or any other Bluetooth-connected device, which resolves the previously reported difficulty that patients experienced with a prior version of the device that required placing a bone conduction piece over the ear.^23^ OrCam Read also eliminates the problem of using the device in low light and locating the buttons, as OrCam Read is voice-activated. The user can direct the device to read the desired portion of the text, instructing the aid to read the entire or half of the page, reducing the need to re-listen to information. The price of the device remains a barrier to access ($1,600–1,999). Future investigation of OrCam efficacy can potentially help provide sufficient evidence for the device to be covered or partially subsidised by the major insurance providers.

Limitations of this study included its small sample size in both study populations as well as the heterogeneity of the type of low vision. We were unable to conduct a paired t-test analysis due to attrition as three patients did not complete the follow-up NEI-VQF-25 questionnaires. We also used a non-validated test to assess daily activity performance and capture patient satisfaction. Although most patients had a positive experience with OrCam, our sample size was too small to capture a meaningful effect in NEI-VFQ-25. However, we did see improvement on several subscales, and we believe that repeating this study with a larger sample of patients can produce a more accurate assessment of the impact of the device on patients’ QoL. Despite the heterogeneity of the conditions that resulted in low vision in participants of our study, low vision is an ensuing disability of both brain tumours and stroke that affects patients of both groups. At the same time, the use of the device may have a different impact depending on the aetiology of low vision, such as central or peripheral. In our forthcoming larger study, we intend to explore the impact of this device for different aetiologies using a standardised, Likert-like scale to assess patient satisfaction and design the assessment of daily tasks based on a composite of previously used function tests in reports evaluating vision devices.

Our study had several strengths. As a feasibility study, we used validated and reliable tools to evaluate the VRQoL in patients with a chronic eye condition. While most studies tend to focus on reading speed as the primary evaluation metric of vision aids, we completed a broad assessment to evaluate the impact of OrCam Read on VRQoL, daily function, and user experience. We also had a unique patient population and a device that had not been evaluated before. While we used validated tools to assess vision-targeted health status, our results and ability to draw conclusions were significantly limited by the small sample size.

In conclusion, this study demonstrated the feasibility of investigating the impact of a portable vision device on VRQoL in patients with visual impairment due to stroke or brain tumours. There was an improvement in daily function and independence following a 1 month intervention with the OrCam Read. This device may allow them to enjoy improved vision-related independence in daily life. A larger study is warranted to validate the study results in these populations.
